# Hospital and Institutional News

**Published:** 1919-08-09

**Authors:** 


### HOSPITAL AND INSTITUTIONAL NEWS.

### HEALTH OF TROOPS ON AFGHAN FRONTIER.

With regard to the allegations recently published as to the defects in hospital management and supplies, it was stated in Parliament that Mr. Montagu is having a special inquiry made into each definite allegation that is brought to his notice. In the meantime, orders have been issued that medical officers are to ask for whatever they deem necessary for the comfort of the sick and wounded in their charge and that their demands are to be met at once. Additional hospitals for 4,000 British and 8,000 Indian troops have been established in specially fitted barracks in proximity to the frontier, and electric lighting and fans where none already exist are being supplied to the former. Convalescent depôts for officers and soldiers have, in addition, been formed and special arrangements have been made for the supply of fresh milk to the sick in hospital, and cows for this purpose have been placed so far at the front of Dakka, Bannu, and Tank. The scale of equipment of Indian general hospitals has been reviewed and arrangements for providing additional equipment are in progress. The Indian authorities are evidently awakening to a sense of responsibility and of appreciation of the requirements of the situation.

### SUBSIDIES FOR MEDICAL EDUCATION.

The *British Medical Journal* announces that the Government is offering subsidies to four leading medical schools, if certain conditions are complied with. These grants, it is stated, are to go towards salaries for whole-time members of the hospital staff, who shall devote a proportion of their time to teaching. It is only a few months ago that this innovation of well-paid resident physicians and surgeons on an equal footing with the visiting staff was introduced by one or two pioneer institutions, and misgivings were expressed at the time as to where the money was to come from. It looks as if the managers of the hospitals concerned had very intelligent foresight of the action the Government were likely to take, or else had acted upon a hint from authority.

### PLAGUE IN LIVERPOOL.

A fatal case of bubonic plague has occurred in Liverpool. The patient, a master stevedore and bargeman was removed to the Isolation Hospital on 11th inst. and died on the 19th inst., but bacteriological confirmation of the fact that he was suffering from plague was not obtained until 26th inst. No further cases have occurred. Dead rats have been discovered in the patient's office and in the adjacent premises, but on examination, none were ascertained to have plague. A mouse found dead has been proved by bacteriological examination to have suffered from plague. The Corporation have undertaken the disinfection of the premises and the destruction and examination of rodents found in them. One of the Medical Officers of the Ministry of Health is engaged on a detailed investigation of all the circumstances of the case.

### EARL SPENCER'S GIFT TO NORTHAMPTON.

At the Quarterly Court of the Northampton General Hospital it was reported that Earl Spencer had given Dallington House and five acres of ground to the hospital, to be used as a convalescent home. The conveyance of this important estate has been made to trustees, and should at any future time it no longer be required for convalescent home purposes, it shall be held in trust for such charitable purposes as the trustees may decide. The question of maintenance is substantially met by an offer of the Prisoners of War Fund Committee to give £25,000, from their surplus funds, to the home. Part of this sum—£5,000 is suggested—is to be used for the necessary alterations to Dallington House, and the balance is to be used towards upkeep. It is expected that forty beds will be established—twenty for male and twenty for female patients, who will only be admitted to the home from the hospital, priority being given as far as possible to ex-Service men. The hospital has had a very valuable gift, through the Demobilisation Board of the V.A.D., of the operating-theatre and X-ray apparatus from the Barry Road Hospital. The V.A.D. are also paying for taking down the building at Barry Road and re-erecting it in the grounds of the hospital.

### THE USE OF INDIAN OPIUM IN EUROPEAN MEDICINE.

In pre-war times the opium used in this country for medicinal preparations and for the manufacture of morphine was obtained from Turkey and Persia. It was at one time believed that Indian opium was not rich enough in morphine to be employed for these purposes. Although this is true of a large part of the opium prepared in India for export to the East, it is now clearly established that opium suitable in every way for medicinal use in Europe and for the manufacture of morphine can be readily obtained from certain areas in India. So long ago as 1896 the Imperial Institute suggested to the Government of India that the production of medicinal opium for export to Europe should be undertaken. No action was however taken in this direction until 1907, when the question was again considered at the suggestion of the Imperial Institute in connection with the restrictions which were then placed on the future export of Indian opium to China. Finally, after the outbreak of war, the Government of India permitted the export of a certain quantity of opium to the United Kingdom for use by manufacturers of morphine, and it is hoped that the trade thus begun will be developed and firmly established. The proof that Indian opium is of much better quality than was previously supposed is mainly due to investigations carried out at the Imperial Institute. In a detailed report published in the *Bulletin* of the Imperial Institute in 1915 it was shown that of 102 samples of opium from different parts of India about half could be substituted for the best Turkey and Persian opium employed in European medicine, whilst an additional 25 per cent. could be used for special medicinal purposes for which a smaller proportion of morphine in the opium is sufficient. The results of further investigations, which are published in the current number of the *Bulletin*, confirm the earlier opinion as to the high quality of Indian opium. The average amount of morphine in twenty-four samples from various parts of the United Provinces was found to be above the highest standard demanded by the British Pharmacopœia. In fact nineteen of them were so rich in morphine that they would need dilution with lower-grade opium before they could be used for medicinal purposes in the United Kingdom. Similar results were obtained in the case of twelve samples of Benares opium, representing the different kinds available for export, all of which were found to be quite suitable for medicinal use or for manufacturing purposes in the United Kingdom.

### A VENEREAL DISEASE PROBLEM.

Should early preventive treatment be provided in connection with the campaign against venereal disease? The L.C.C. Public Health Committee thinks not. It has had a deputation from the National Council for Combating Venereal Diseases, which urged the municipality to arrange for early treatment at approved centres within a short time after exposure to possible infection. In a very careful discussion of the advantages of early preventive treatment, it was stated by the deputation that, in the case of syphilis, six hours was the maximum period, after exposure to infection, within which such treatment could be effective. Hence, as the Public Health Committee now reports, for successful results to be achieved from early treatment a very large number of centres, surgeries, etc., would have to be provided in all parts of London. Moreover, with one exception, hospitals cannot arrange for the introduction of early treatment. The committee concludes that, apart from the moral questions involved, the medical advantage in particular cases from the introduction of early preventive treatment would probably be nullified by a consequential increase in the number of cases of exposure to infection.

### DOCTORS FOR THE R.A.F.

The Air Ministry announces that the R.A.F. requires the services of a certain number of medical practitioners, including those who have already served. Candidates must be under 35, fit for general service at home or abroad and willing to fly if called upon to do so. If they have served before, they will be commissioned in their previous substantive rank, otherwise they will be lieutenants. Engagement is to be for one year, or until no longer required, whichever shall first happen. Pay will be at the rate of £550 a year, inclusive of all allowances, except travelling allowances and expenses when travelling on duty, and rations or the allowance in lieu. Outfit and kit allowance will be issued to candidates who have not received them for previous service. Applications should be addressed to the Secretary, Medical Dept., Air Ministry, London, W.C. 2.

### ACCOUNTS AND COST RECORDS.

The Agricultural Costings Committee will be shortly issuing a series of pamphlets dealing with farmers' accounts and cost records in view of the importance of these subjects and the need of their being taken up by the industry. Farmers and others who are already in communication with the Committee will receive these, and any other agriculturists desirous of having copies sent from time to time should notify the Director of Agricultural Costs, Palace Chambers, Westminster, S.W.1. The pamphlets will deal with the advantages to be obtained from proper account-keeping as distinct from cost-keeping, and descriptions of suitable and simple systems of book-keeping will be given. The more involved field of cost-keeping and its difficulties will also be discussed. Various methods of keeping accounts will be dealt with and explanations and advice given, not only relating to simple accounts but also with regard to more complete systems such as would be required, say, on the larger and more progressive farms. The various methods of ascertaining costs will be outlined, the books and records necessary for the purpose will be dealt with, and the problems and questions of principle involved in "Costing" will be discussed.

### "EMERGENCY" HEALTH.

Panic regulations are not always conducive to good, but those made during the influenza epidemic to secure the adequate ventilation of picture palaces proved beneficial, and local authorities asked that such regulations should be made permanent. Nevertheless, the Local Government Board—now the Health Ministry—has made an order rescinding the regulations. Now some of the London Borough Councils are agitating for the renewal of the regulation, their contention being that they were not only effective in preventing the spread of influenza, but also of other infectious diseases. Hence representations are now being made for the renewal of the regulation in the interests of the public health generally.

### THE KING AND WALES.

The Welsh National Memorial Association is understood to have invited His Majesty King George next spring to visit and open the New Sanatoria at Llangwyfan, for South Wales, and at Pontywal, Talgarth, for South Wales, which it has established to complete the scheme that it was founded to promote. Major David Davies, M.P., who founded the Association under the inspiration of King Edward, in reviewing the work accomplished believes that sufficient has been done to warrant a request being made to the King for the honour of his inspection. Apart from the buildings erected, Major Davies points to the research undertaken, and it is suggested that the Ministry of Health is the outcome of the movement which first expressed itself in the anti-tuberculosis campaign. This, of course, was started in Wales on ambitious lines; it became the Principality's memorial to King Edward, and though the Association has had many difficulties, Major Davies believes that much has been done in prevention, treatment, and research. To complete the work, His Majesty's visit would, of course, give the final stimulus. But in these days, when the claims on the Royal Family are unusually severe, it would be unwise more than to hope that the King may find himself able to pay a visit. The cause is his own, but his time is his people's, and he cannot be everywhere at once.

### QUEEN CHARLOTTE'S APPEAL.

QUEEN CHARLOTTE'S Hospital has suffered severely during the war by reason of its income falling far short of the greatly increased expenditure. It is therefore forced to appeal for upwards of £10,000 to make up the deficiency. A number of generous contributions have been received, including the following:—Lord Portman £1,000, De Beers Consolidated Mines £500, Anonymous £250, Alex. B. Williamson, Esq., £250. A sum of £800 has also been received from the concert arranged by Adele Lady Cadogan, Lady Lowther, and friends held recently at Chelsea House kindly lent by Lady Philipps. An urgent appeal for the balance of nearly £8,000 is now made. The cost of maintaining the hospital last year was about double what it was before the war, which means that an additional £7,000 must be raised annually. In addition the Committee are anxious to proceed at the earliest possible date with the scheme of extension and improvements which had to be postponed during the war, the cost of which is now estimated at upwards of £40,000. The Treasurer (Mr. Anthony de Rothschild) will gladly acknowledge contributions sent to him at the hospital, Marylebone Road, N.W. 1. During the war the hospital has either received into the wards or attended in their own homes over 5,000 soldiers' and sailors' wives.

### HOSPITAL NEWS IN LOCAL PAPERS.

ASKED to define the quality that makes a good journalist, a distinguished editor said: "A good journalist is he who, when sent to describe a cricket match, and prevented, because of the crush, from seeing more of the game than was visible between the feet of the man in front, will write an article on "Seeing a cricket match between another fellow's feet." This power of having your pen on the object, even if the object is not the one you intended, is necessary for the hospital man who writes in local papers. He will desire to have hospital news in each, and if possible the news should appear differently. It requires considerable skill and energy to keep intimate relations with newspapers, because the life of a lay newspaper is usually a matter of hours. We are beginning to see an expansion of hospital journalism. Secretaries feed the papers better than they once did, and reap their reward accordingly. But we have yet to see the busy secretary of a large hospital write a weekly *causerie* about his institution; and yet how a hospital abounds in subjects, in humour, in the thousand interests that go to make these essays in little! It would be child's play, we imagine, for an essayist like Mr. E. V. Lucas. He is a model of fecundity and fancy, and a tithe of his talent would fill a column in a newspaper about a hospital throughout the year. This talent cannot be so rare as the absence of this column about hospital life leads us sometimes to fancy. Where does it hide?

### THE CHELMSFORD SCHEME.

THE centenary of the Chelmsford Hospital is being used to launch an important scheme. The hospital at present is overcrowded, the nurses' quarters are poor, and more beds are needed for the district. It is intended to increase the number from forty-three to eighty, and, fortunately, four years ago the first step was taken to this end. Mr. C. E. Ridley then began to collect £4,000, most of which has been raised, and the plan now is to secure £20,000, so as to meet the cost of the later scheme of enlargement. About half is in hand or has been promised. It is necessary to have a surer income than has been the case of late, when the situation has been saved by the receipt of comparatively large sums from unexpected quarters. So far, however, no wide attempt seems to have been made to organise subscriptions from each section of the population, urban and rural, employers and employed. This, we believe, is the one solution of the maintenance difficulty, and the Hospital Committee must adopt a policy of the kind at latest when the capital sum now asked for has been reached. Its chief requisite is energy and an up-to-date resourceful energetic organiser would revel in the opportunity Chelmsford offers to such a man or woman to do a good work and win much reputation for the individual and the hospital. Unlike Colchester, Chelmsford can be energetic when it chooses and win victory, whatever the difficulties. The extension, it is true, may enable the hospital to be paid for some classes of patients under the Minister of Health, but he who banks on that forgets the red tape with which such grants might saddle him.

### THE FOUR-HUNDRED COMMITTEE.

IN this connection it is interesting to note the Cardiff Hospital's instrument for electing its honorary medical staff. It illustrates how improved administration may be found even under the most unsatisfactory conditions, when there is an enlightened determination to find them. The constitution of the hospital management is extraordinary, composed of an elective element hopelessly outnumbered by Presidents and Vice-Presidents, whose minimum qualification is an annual subscription of ten guineas, contributed individually or reflectively through a representative. Hence there are probably 400 members of the Board of Management. The collective wisdom of this body is that, recognising its unfitness to discriminate the technical merits of medical candidates on its own initiative, it followed our scheme of an Election Committee of twenty-one. The composition of this Committee is as follows:—The Chairman of the Board of Management, the Chairman of the various Sub-Committees (5), the Honorary Treasurer of the Hospital, the Principal of the University College of South Wales and Monmouthshire, the Dean of the Medical Faculty of the University College of South Wales and Monmouthshire, and twelve members of the Board of Management, elected by the Board of Management at their usual monthly meeting in April of each year. This once more reminds us that cumbersome representative machinery is not to be mistaken for democracy, which, as it is apt to will a dictator in an emergency, is also most workable when it delegates particular tasks.

### MESS AND METHOD AT CARDIFF.

Sometimes in the life of an institution many changes take place at or about the same time. It looks as if the changes were related and yet we know that each has happened independently. At the King Edward VII. Hospital, Cardiff, the deaths of Colonel Bruce Vaughan and other lay friends of the hospital synchronise with important changes in the Honorary Medical Staff. The Senior Surgeon, Mr. Rhys Griffiths, who would have retired about the beginning of the war, but by the Board's request continued in office, has now withdrawn from the active staff and becomes a Consultant, and Colonel Herbert G. Cook, the Senior Assistant Surgeon, has been elected a surgeon of the hospital. Colonel Cook had just returned after three years' service as Officer Commanding the Welsh Hospital at Netley. Previous to that Colonel Cook had been in command of the Glamorgan and Monmouthshire Hospital, in service with the French Army. Colonel A. W. Sheen (who became Senior Surgeon to the Hospital upon Mr. Rhys Griffiths' resignation), the first Officer Commanding the Welsh Hospital at Netley, had just been released from a command in India, when he accepted the office of Consulting Surgeon to the Ministry of Pensions in the London area.

### WHITWORTH HOSPITAL FIASCO.

We cannot believe that the closing of the Whitworth Hospital, one of the three Homes of Industry in Dublin whose plight we have already mentioned, will be the end of the matter. It closed on the 26th ult., and its fellow-institutions the Hardwicke and the Richmond cannot be much better off. We understand that the firms which have been supplying the Whitworth Hospital have received no payment since February last. Many of the nurses who were receiving their training were sent to their homes a few weeks ago, and the extern departments have been closed for a similar period. The absence of any general support, apart from the Government grant of £6,400 to the board of the three institutions, is now complained of; but it is only a few years since the total income was considerably larger, so that the inference is that the public and the patients have ceased to do their share. The total income during recent years is recorded in Burdett's "Hospitals and Charities," and it would be sinister indeed if state aid, which is being invoked in this country were, in time, to reduce voluntary contributions over here to vanishing point. Frankly, no hospital can be allowed to be closed at present, and we await the effort which necessity imposes for the prompt re-opening of the Whitworth Hospital.

### "TO WIT"—TO WHICH?

A brief record of the work of the auxiliary military hospitals in Middlesex since 1914 has been compiled by Clive Fenn and published by the Saint Catherine Press, price 1s. It is not so much a history as a tribute, with documents, in verse and prose by several people. Sir Owen Seaman writes some graceful introductory stanzas; the Duchess of Northumberland gives her benediction to the enterprise, and there follows a series of impressions of the work at Hampton Court, of the life of an ambulance officer, of that august figure of the sergeant; even a patriotic grievance is included. The county is the unit described, and the share of private enterprise in the work is inspiring to read. In all, over 2,500 beds have been supplied within its area, and the list of hospitals appended is a valuable record. There are also various sketches, half story, half reminiscence, to illustrate the good fellowship or patriotism of the different classes of war worker volunteers. In the main, "Middlesex to Wit," for such is the title, may be expected to appeal to those who took part in its record, but there is perhaps more for the public, and our only criticism is the question whether it would not have been wiser to limit the "Wit" to one or the other audience.

### COTTAGE HOSPITALS AND CENTRAL CONTROL.

Margam Cottage Hospital, which was founded and equipped by the late Miss Talbot, has now passed under the control of the Port Talbot and Aberavon Hospital. The maintainer of the Margam estate says that he is too much taxed to continue to support the hospital, which has been reopened as a branch of the Aberavon. The twelve beds which it contains will be a useful addition to the former hospital.

### HONOUR FOR A HOSPITAL PHARMACIST.

Mr. C. H. Hampshire, B.Sc., F.I.C., has been appointed Hon. General Secretary of the British Pharmaceutical Conference, to succeed Mr. Horace Finnemore, who, until recently, was chief pharmacist at Guy's Hospital, and has now entered the wholesale drug trade. Mr. Hampshire, who is well known as chief pharmacist at University College Hospital, had a distinguished career as a student at the Pharmaceutical Society's School of Pharmacy, obtaining several medals in competition. He is also an expert on volumetric analysis, on which he has published a text-book. It is interesting to note that Mr. R. R. Bennett, B.Sc., who shares the secretarial work of the Conference with Mr. Hampshire, was formerly a hospital pharmacist, and has contributed valuable papers on the practice of pharmacy to the Conference. The announcement was made that next year's Conference is to be held at Liverpool, where it was held some few years ago.

